# Bio-Based Hydrogels Composed of Humic Matter and Pectins of Different Degree of Methyl-Esterification

**DOI:** 10.3390/molecules25122936

**Published:** 2020-06-25

**Authors:** Assunta Nuzzo, Pierluigi Mazzei, Davide Savy, Vincenzo Di Meo, Alessandro Piccolo

**Affiliations:** 1 Centro Interdipartimentale di Ricerca sulla Risonanza Magnetica Nucleare per l’Ambiente, l’Agroalimentare ed i Nuovi Materiali (CERMANU), Università di Napoli Federico II, Via Università 100, 80055 Portici, Italy; davide.savy@unina.it; 2 Dipartimento di Farmacia (DIFARMA), Università di Salerno, Via Giovanni Paolo II 132, 84084 Fisciano, Italy; pmazzei@unisa.it; 3 Dipartimento di Agraria, Università di Napoli Federico II, Via Università 100, 80055 Portici, Italy; vincenzo.dimeo@unina.it

**Keywords:** hydrogels, humic substances, compost, release, low and high degree of pectin methylation, NMR, SEM, MRI, rheology, lignocellulose biorefinery waste

## Abstract

We prepared humo-pectic hydrogels through ionotropic gelation by crosslinking natural pectins of different degree of methyl-esterification with either humic substances (HS) extracted from cow manure compost or humic-like substances (HULIS) from depolymerized lignocellulose biorefinery waste. The hydrogels were characterized by solid-state ^13^C-NMR spectroscopy, scanning electron microscopy, spectroscopic magnetic resonance imaging and rheological analyses. Their ability to work as controlled release systems was tested by following the release kinetics of a previously incorporated model phenolic compound, like phloroglucinol. Our results indicated that the release properties of hydrogels were influenced by the molecular composition of HS and HULIS and by the different degrees of methyl-esterification of pectins. The hydrogel made by the high methoxyl pectin and HS showed the fastest rate of phloroglucinol release, and this was attributed not only to its morphological structure and crosslinking density but also to the least formation of ionic interactions between phloroglucinol and the polysaccharidic chains. Our study suggests that the efficiency of novel humo-pectic hydrogels as sustainable carriers of agroproducts to crops is related to a careful choice of the characteristics of their components.

## 1. Introduction

In recent years, natural polymers have attracted increasing attention due to their natural abundance and widespread applications as biomaterials. Pectins are biodegradable and biocompatible polymers located in the primary cell wall and middle lamella of many plants [1]. They are widely used in the pharmaceutical and food industries for a large number of applications, such as gelling agents, thickeners, stabilizers and emulsifiers [2,3], as well as carriers for nasal, ocular, and oral drug delivery [4,5,6,7] and wound healing [8]. Commercially, most pectins are obtained from citrus fruits like orange, lemons, grapefruit, and apples [9,10], which are the abundant residues of juice productions.

Pectins are heterogeneous polysaccharides with three main structural domains: homogalacturonan (HGA), alternating with two types of highly-branched rhamnogalacturonan regions designated as rhamnogalacturonan-I (RG-I), and rhamnogalacturonan-II (RG-II) [11,12]. HGA is the major component of pectic polysaccharides and consists in linear polymers, mainly composed of α-(1→4)-D-linked galacturonic acids [13,14,15] that can be partially methyl-esterified and sometimes partially acetyl-esterified [16]. According to their degree of methyl esterification (DE), pectins can be classified as low methyl-esterified pectins (LMP) (DE < 50%) or high methyl-esterified pectins (HMP) (DE > 50%) [17,18]. One of the main attractive characteristics of pectins is their capacity to form gels. The gel-forming ability of pectins increases with the decreasing degree of methylation. LMP can gel in the presence of divalent cations, usually calcium (Ca^2+^) [19], and the gelation is due to the formation of intermolecular junction zones between pairs of carboxyl groups in the homogalacturonic smooth regions of different chains in close contact [20]. The structure of such a junction zone is generally attributed to the so-called “egg box” binding process [21,22]. In HMP systems, instead, junction zones are stabilized mainly by hydrogen bonding between carboxyl and secondary alcohol groups and by hydrophobic interactions between methyl esters [23].

In a previous work, we used low methyl-esterified pectin for a low-cost and environmentally-sustainable production of novel formulations of hydrogels containing either HS extracted from different composted biomasses or HULIS from depolymerized lignocellulose waste of biorefinery activities [24]. Such novel humo-pectic formulations were aimed to function as carriers of bioactive compounds for the controlled and safe delivery of agroproducts to plant systems. However, they could also directly act as plant biostimulants because of the presence of bioactive humic substances and lignocellulose residues. In fact, both HS from compost and HULIS from biorefinery lignocellulose residues were found to be active as plant biostimulants [25,26], and their application to soil was shown to improve soil physical and chemical properties, crop production [27], and macronutrient availability [28]. HS and HULIS are regarded as supramolecular associations of heterogeneous and relatively small molecules, which are held together in only apparently large molecular sizes by weak forces, such as hydrogen and hydrophobic bonds [29], and whose conformations can be disrupted by the action of weak organic acids [30], thereby allowing the release of organic bioactive molecules and nutrients [31].

The aim of this work was to investigate the effects of HS and HULIS and the methylation of pectin carboxyl groups on the diffusion properties of active compounds out of hydrogels (H) obtained through ionotropic gelation by Ca^2+^ ions. The hydrogels were formed with pectins of different degrees of methyl esterification in combination with either HS extracted from cow manure compost or HULIS from depolymerized lignocellulose waste of biorefinery activities. The microstructure and the rheological properties of hydrogels were also studied, and so was the ability to release a model phenolic compound from these humo-pectic hydrogels.

## 2. Results and Discussion

### 2.1. ^13^C CPMAS NMR Spectroscopy

**Figure 1:**
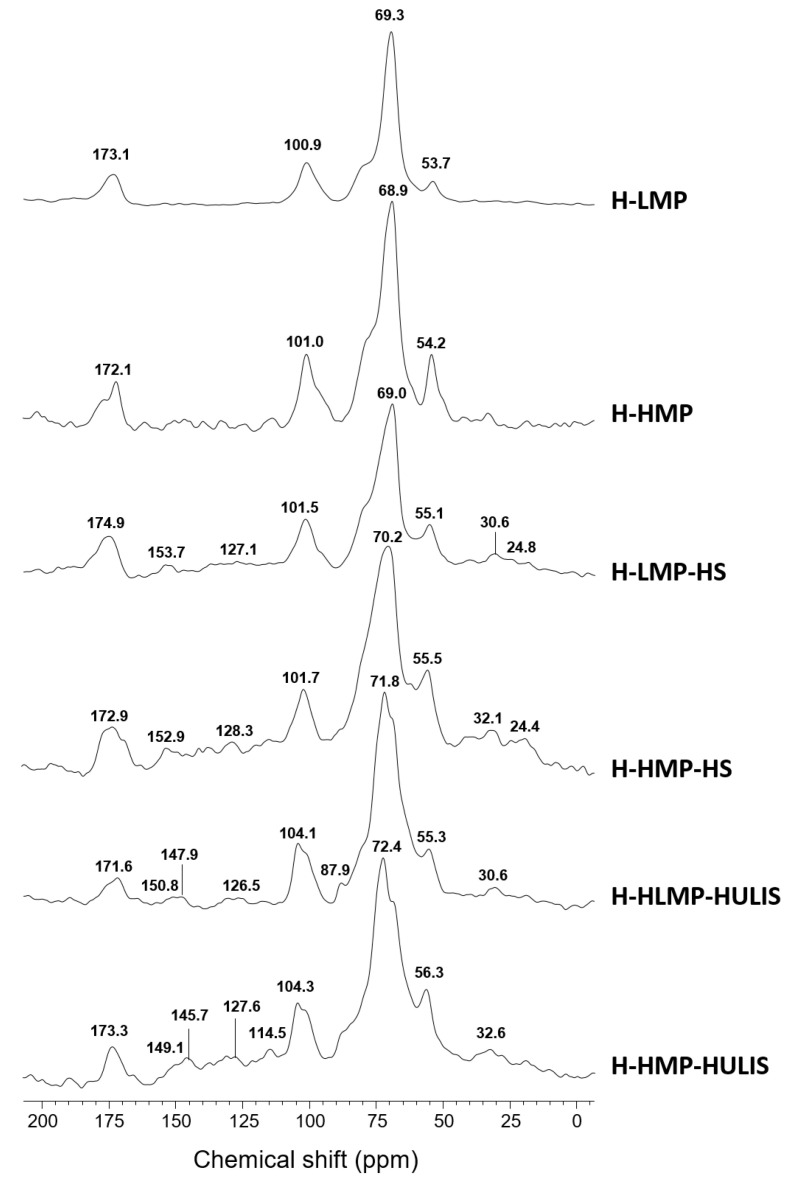
^13^C CPMAS NMR spectra of hydrogels based on low methyl-esterified pectin (H-LMP), high methyl-esterified pectin (H-HMP), or on their combination with either humic substances extracted from cow manure compost (H-LMP-HS and H-HMP-HS) or humic-like substances from depolymerized lignocellulose biorefinery residues (H-LMP-HULIS and H-HMP-HULIS).

The ^13^C CPMAS NMR spectra of hydrogels are shown in Figure 1. Both spectra of H-LMP and H-HMP showed a signal around 173 ppm due to C-6 carbons of galacturonic units and a band at 101 ppm derived from the anomeric C-1 carbon. The resonance at about 69 ppm was assigned to the other carbons of pyranoid ring, while the signal around 54 ppm represented methyl carbons of the COOCH_3_ methyl ester [32]. Similarly, the solid-state NMR spectra of humo-pectic hydrogels showed a predominance of hydroxylated carbons (60–110 ppm), contained in either the carbohydrates of the residual cell wall or lignin lateral chains. The signal centered at 55 ppm was attributed to methoxyl substituents in aromatic rings of guaiacyl and syringyl units in lignin, and, partially, to C-N groups in peptides [33]. Moreover, the resonances over the alkyl-C interval (0–45 ppm) suggested the presence of methylenic (CH_2_) chains in various lipid molecules, such as wax and cutin, and CH_2_ groups in β position to carbonyls in aliphatic esters. The signals in the 110–145 ppm interval derived from unsubstituted phenyl carbons in either lignin monomers, lignans, flavonoids or unsaturated lipid structures, while those in the phenolic region (145–160 ppm) were indicative of O- and C- substituted aromatic structures, pertaining to lignin and lignan components. Finally, the intense signal in the carbonyl region (160–190 ppm) at about 172 ppm referred to carboxyl and amide groups in aliphatic acids and peptides, respectively [24].

Spectral integration revealed a composition dominated by carbohydrates (60–110 ppm) for all hydrogels, but H-HMP, H-HMP-HS and H-HMP-HULIS showed a smaller content of carboxyl groups (190–160 ppm) and a larger presence of methoxyl substituents (60–45 ppm) than H-LMP, H-LMP-HS and H-LMP-HULIS (Table 1). These differences in hydrogels are mainly a function of the greater methylation percentage in HMP. Moreover, the greatest hydrophobicity index (HB/HI) shown by the hydrogel prepared with HMP and HS extracted from manure compost should be attributed to the hydrophobic affinity of both its humic aryl and alkyl components with the greater degree of methylation of carboxyl groups in HMP (Table 1). The lesser hydrophobicity of the H-HMP-HULIS sample may be due, instead, to the absence of alkyl components in the HULIS materials that prevented a similar binding affinity with the high methyl-esterified pectin, like that shown by HS (Table 1, Figure 1). These observations are in line with the supramolecular model of humic substances that depicts humus as a self-assembling association of relatively small heterogeneous molecules held together by weak forces [29,34], whose stability responds to chemical changes such as concentration, pH, ionic strength, and metal content [31,35].

### 2.2. Morphology of Hydrogels by SEM and MRI Analyses

**Figure 2:**
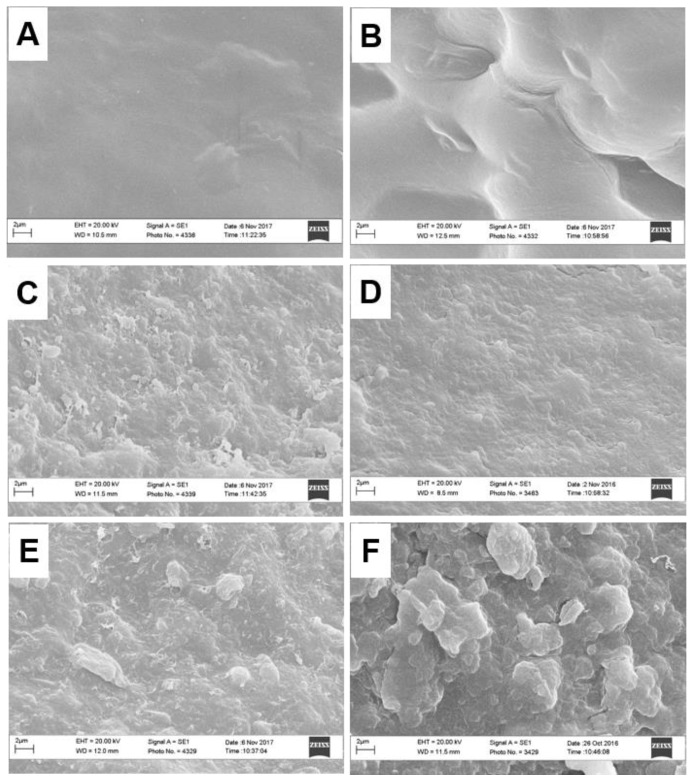
SEM micrographs of freeze-dried hydrogels: (**A**) H-LMP, (**B**) H-HMP, (**C**) H-LMP-HS, (**D**) H-HMP-HS, (**E**) H-LMP-HULIS, (**F**) H-HMP-HULIS.

The morphological structure of hydrogels was characterized by scanning electron microscopy (SEM) and spectroscopic magnetic resonance imaging (MRI). Figure 2 shows the SEM images of the cross-section of freeze-dried hydrogels. Unlike the images of H-LMP and H-HMP, which revealed a smooth and homogeneous microstructure (Figure 2A,B), the images of humo-pectic hydrogels displayed a rougher and lumpier structure, thereby suggesting that the incorporation of HS and HULIS modified the surface morphology of hydrogels. In particular, H-HMP-HS seemed to have the smallest micrograins dispersed on its surface (Figure 2D).

**Figure 3:**
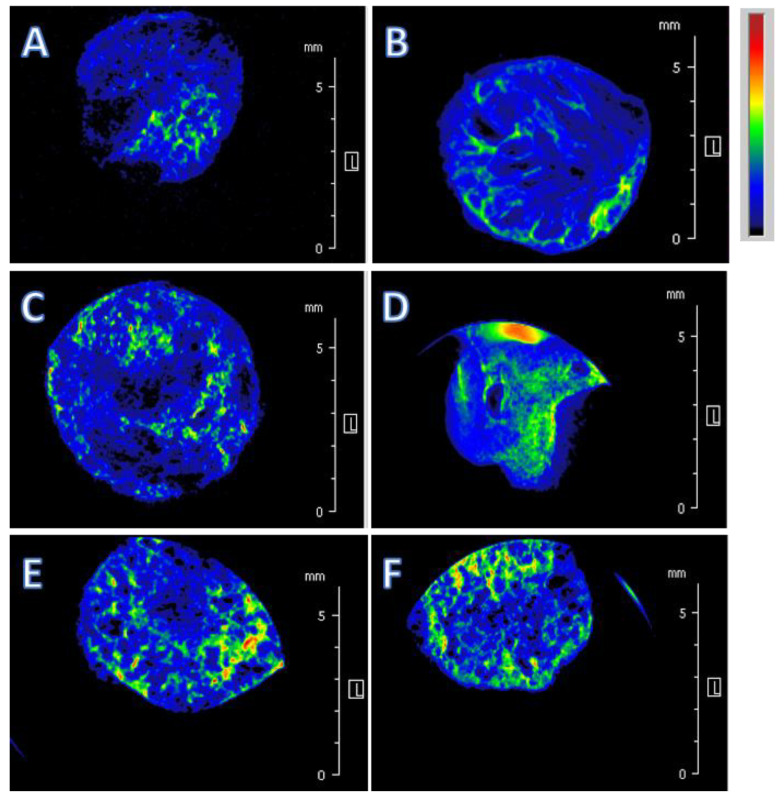
MRI images showing a representative central slice of each hydrogel after the addition of 150 μL of water necessary to saturate samples: (**A**) H-LMP, (**B**) H-HMP, (**C**) H-LMP-HS, (**D**) H-HMP-HS, (**E**) H-LMP-HULIS, (**F**) H-HMP-HULIS. The MRI images show the spatial distribution of water within the structure of samples, varying the color from blue to green and pale red as a function of increasing water content.

MRI spectroscopy was applied in order to obtain further information on the structural characteristics of hydrogels (Figure 3). MRI images revealed that water molecules, after an extensive and uniform distribution in hydrogels (blue colored section), concentrated in air-filled cavities corresponding to pores (green colored spots). The areas reporting an intense response (spots with colors fading toward yellow-red levels) indicated a relatively larger content of free (unbound) water. The observation of the distribution of water molecules in hydrogels allowed for direct detection of their internal structures [24]. The MRI images revealed that H-LMP and H-HMP had similar small porous microstructures (Figure 3A,B), whereas H-HMP-HS showed the largest number of small-size pores diffused within the hydrogel (Figure 3D)

**Figure 4:**
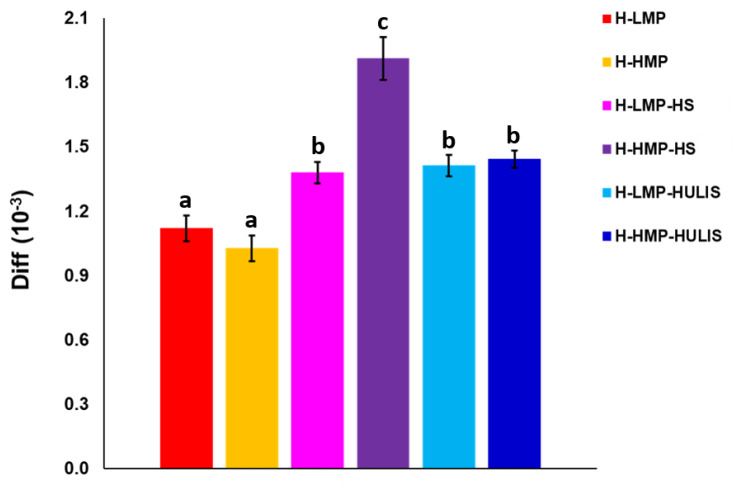
Diffusion coefficients (Diff) obtained from MRI experiments for the different hydrogels after the addition of 150 μL of water necessary to saturate samples. Error bars indicate standard error (n = 3), and different letters indicate significant differences by the Tukey’s test at *p* ≤ 0.05.

Furthermore, MRI experiments for the different hydrogels allowed us to measure the apparent diffusion coefficients (Diff), which enabled an evaluation of the extent of free diffusion of water molecules within the hydrogel matrix. Diff values decrease when water molecules encounter physical barriers to their diffusion, whereas they increase if water can move more freely through a less compact hydrogel (longer free diffusion distance) [24]. H-HMP-HS showed the greatest Diff value (Figure 4), thus indicating a fast diffusion rate of water molecules through this sample, probably because of the large amount of micro-size pores, whereas H-LMP and H-HMP revealed the smallest Diff values, which proved a much more compact structure in these hydrogels and a limited water diffusion capacity.

The results of SEM and MRI analyses indicated that the incorporation of both HS and HULIS into pectic matrices modified the inner structure of hydrogels, including the distribution, size, and shape of pores, which, in turn, affected the diffusion rate of water molecules through the hydrogels.

### 2.3. Rheological Characterization of Hydrogels

Rheological measurements provided information about the viscoelasticity of the different hydrogels. Viscoelasticity was characterized by the storage modulus G′ (proportional to the extent of the elastic component) and loss modulus G″ (proportional to the extent of the viscous component) [36,37]. Hydrogels were analyzed under oscillatory strain sweep tests and the storage modulus G′ of water-hydrated samples was calculated as a function of strain (data not shown). Up to strains of about 3%, the storage G′ modulus was independent of the applied strain for all the investigated systems, thus allowing the identification of the linear viscoelastic region. Since all the rheological experiments should be conducted in the linear viscoelastic region to avoid any disruption of the sample structure [24], a strain of 2% was chosen for the subsequent frequency sweep tests.

**Figure 5:**
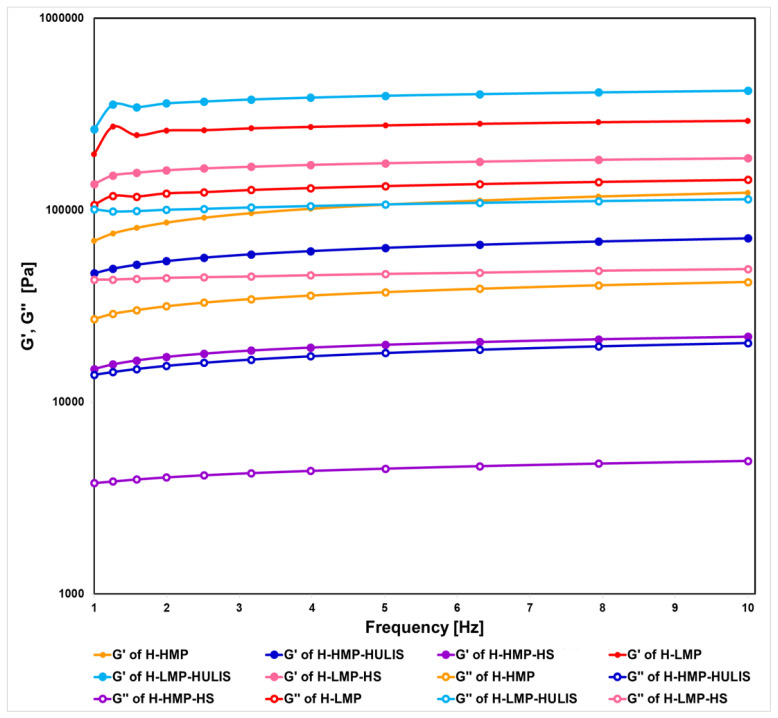
Storage G′ (full circles) and loss G″ (empty circles) moduli as a function of applied frequency.

The dependence on the frequency of G′ and G″ viscoelastic moduli determined for the hydrated samples is reported in Figure 5. In all hydrogel systems, the storage G′ and loss G″ moduli exhibited a similar power-law dependence on the frequency that was expressed by straight lines nearly parallel to each other and slightly increasing with frequency. Moreover, G′ values were greater than G″ ones over the entire frequency range (1–10 Hertz) for all samples, but both viscoelastic moduli were greater in humo-pectic hydrogels prepared with LMP than those with HMP. These findings pointed out that all hydrogels had a solid-like viscoelastic nature, although the LMP-based hydrogels revealed a larger crosslinking density and a greater chain flexibility than those containing HMP. This difference can be explained by the fact that LMP-based hydrogels contained more free carboxyl groups that promoted the formation of egg-boxes structures, which contributed considerably to the building of the hydrogels crosslinking network.

Moreover, the smallest G′ value was observed for the hydrogel prepared with HMP and the HS from cow manure compost, thus resulting in the least resistance to mechanical stresses. A possible explanation may be that not only the abundant methyl groups in HMP but also the alkyl and aromatic structures (Table 1) of humic matter hindered the capacity of polysaccharide chains to form egg-box structural arrangement, thus making the hydrogel poorly resistant to applied stress. These findings showed that the rheological properties of hydrogels depended on both the molecular composition of either HS or HULIS and the degree of methyl-esterification of pectins.

### 2.4. Phloroglucinol Release Study

**Figure 6:**
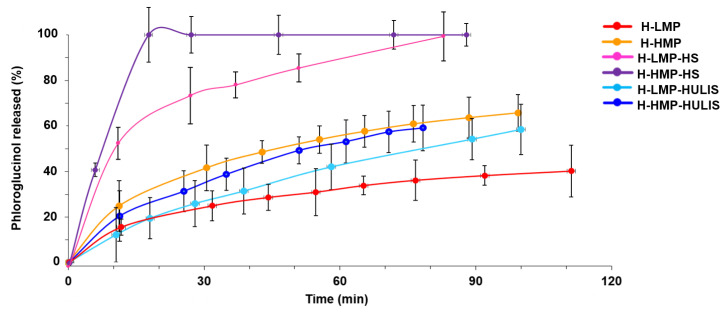
Percent of phloroglucinol released with time from hydrogels in aqueous medium.

The effects of HS and HULIS molecular composition and of the different degree of pectin methylation on the release of bioactive compounds from hydrogels was studied by following the rate of diffusion of phloroglucinol out of hydrogels. Phloroglucinol was used as a model molecule for phenols with plant biostimulation properties. For hydrogels made with HS and either HMP or LMP, the release rate of phloroglucinol was greater than for any other sample, even showing a fast initial phloroglucinol release that was more evident for H-HMP-HS than for H-LMP-HS (Figure 6). In particular, the release percentage of phloroglucinol reached 100% within 20 min for H-HMP-HS and within 85 min for H-LMP-HS. On the contrary, both H-HMP and H-LMP and hydrogels obtained by combining pectins with HULIS exhibited progressive kinetics, whereby the phenolic molecule was slowly released without the initial fast loss. Nevertheless, the release rate was greater for the HMP-based hydrogels than for the ones prepared with LMP. In fact, the percent of phloroglucinol released after about 80 min from H-HMP and H-HMP-HULIS was approximately 60%, whereas the amounts lost from H-LMP-HULIS and H-LMP were approximately 50% and 35%, respectively.

The data obtained by measuring phloroglucinol release from hydrogels were used in the Korsmeyer-Peppas mathematical model [38,39] and the relative kinetic parameters are shown in Table 2. The n values for H-LMP-HULIS and H-HMP-HULIS ranged between 0.5 and 1 and indicated a phloroglucinol release mechanism based on both diffusion and relaxation. In fact, after an initial rehydration with a massive fluid entrance that caused both relaxation and rearrangement of the network of these hydrogels, a more static equilibrium was reached due to synchronization of swelling, Fickian diffusion, and dissolution of phenolic molecule across the hydrogels [39]. For H-LMP and H-HMP samples, the n values were close to 0.5, indicating a Fickian diffusion mechanism for the phloroglucinol release from these hydrogels. Finally, for H-LMP-HS and H-HMP-HS, the n values considerably smaller than 0.5 suggested that phloroglucinol was released by a pseudo-Fickian diffusion. Deviations from the ideal Fickian diffusion may be explained by the erosion of the hydrogel structure due to a dissolution of HS molecules in the aqueous solution [24].

The release of the phloroglucinol from hydrogels may have been affected by different factors. First of all, phloroglucinol molecules may form hydrogen bonds or other ionic interactions with the polysaccharidic chains, thus being more extensively retained in hydrogels [24]. This phenomenon appeared to be larger for hydrogels made by pectin only and by pectin combined with HULIS than for those containing pectin and HS (Figure 6). A reason for this different behavior may be due to the greater content of carbohydrates in the former hydrogels that resulted in a slower and prolonged phloroglucinol release (Table 1). Similarly, the larger degree of pectin methylation in the HMP-based hydrogels may have hindered the formation of hydrogen bonds or other ionic interactions with the phloroglucinol hydroxyl groups, thereby causing a more rapid release of the model phenol than for the LMP-based hydrogels. Moreover, the structural characteristics and the viscoelastic properties of hydrogels may have also affected the release of phloroglucinol from hydrogels. In fact, H-HMP-HS was shown to possess the largest presence of micropores in its structure and the least crosslinking density, thus allowing an easier diffusion of water molecules in its matrix, and, consequently, the fastest release of phloroglucinol.

## 3. Materials and Methods

### 3.1. Materials

Low methyl-esterified pectin (Unipectin OF 300 C; DE = 30%) from citrus was kindly provided by Cargill France SAS in a dry powder form. High methyl-esterified pectin from citrus peels and all other reagents of Reagent Grade were purchased from Merck KGaA (Darmstadt, Germany). The lignocellulose residues were provided by Versalis SpA (Alessandria, IT). These were obtained by applying a hydrolyzing technology on the giant reed (*Arundo donax* L.) that causes a release of cellulose through a microbial enzyme treatment [40]. After the enzyme hydrolysis, the lignocellulose solid residues were separated from the cellulose-rich hydrolysate, extensively washed with deionized water until ion-free, and freeze-dried.

### 3.2. Composts and Extraction of Humic Substances

The manure compost of this study was produced in the composting facility at the Experimental Farm of the University of Napoli Federico II, located in Castel-Volturno (CE). Composting was based on a 4 × 6 m static pile with an air insufflation system composed by three perforated rubber tubes of 10-meter length placed at the bottom of the compost pile and fed by a rotative compressor pump. The composting pile consisted of cow manure (70% *w*/*w*) added with maize straw and poplar trimming as structuring woody materials (30% *w*/*w*). This mixture was uniformly spread by a manure-spreader machine over the insufflation rubber tubes. The composting process lasted 100 days, with periodic monitoring of temperature (5 min interval) and percent oxygen (insufflations flush every 60 min interval) inside the pile. During the first 50 days, the minimum percentage of oxygen insufflated by the air compressor was set at 10%, and thereafter at 5% [26].

HS were extracted from the manure compost as described elsewhere [26]. Briefly, 200 g of air-dried compost was suspended in 1 L of 1 M KOH solution in a polypropylene container and shaken overnight in a rotatory shaker. The supernatant containing humic matter was separated by centrifugation for 20 min at 7000 rpm, filtered through a Wathman 41 filter, brought to neutral pH using 1 M HCl, dialyzed until Cl-free against distilled water, and freeze-dried.

### 3.3. Depolymerization of Lignocellulose Residues

1 g of lignocellulose waste was suspended in 25 mL of distilled water. 350 mg of KOH and 750 μL of a 30% H_2_O_2_ (*v*/*v*) solution were added to the suspension and the reaction mixture was stirred for 30 min at room temperature. Then, the mixture pH was lowered to 6 with a 0.1 M HCl solution, and, finally, the reaction mixture was dialyzed and freeze-dried.

### 3.4. Preparation of Hydrogels

Firstly, 100 mg of LMP and HMP pectin powder were dissolved in 1 mL of distilled water under stirring and mild heating overnight. Also, 5 mg of phloroglucinol and 100 mg of either HS or HULIS from depolymerized lignocellulose biorefinery residues were then dissolved in 1 mL of distilled water by stirring to form a homogeneous solution. After complete dissolution, the LMP and HMP solutions were mixed with the solution containing phloroglucinol and either HS or HULIS at room temperature. The mixtures were kept under fast stirring at room temperature until a jelly-like consistency was reached and then transferred into a watch glass. The materials were then quickly immersed into a 2 M calcium chloride solution to allow the intermolecular crosslinking, and, after an overnight drying at room temperature, were freeze-dried. Control hydrogel samples were prepared as described above but without the phloroglucinol addition.

### 3.5. Characterization of Hydrogels

#### 3.5.1. Solid-state NMR Spectroscopy

The ^13^C NMR spectra were acquired with a 300 MHz (7.0 Tesla) Bruker Avance magnet (Bruker BioSpin, Rheinstetten, Germany) composed by a wide-bore system and equipped with a CPMAS (Cross-Polarization Magic-Angle-Spinning) probe, working at the ^13^C and ^1^H frequencies of 75.47 and 300.13 MHz, respectively. Solid samples (150 mg) were ground, loaded into 4mm zirconia rotors, closed with KelF caps, and spun at a rate of 10,000 ± 1 Hz. The ^13^C NMR spectra were obtained through the CPMAS experiment that consisted in 1814 time domain points, a spectral width of 300 ppm (22,727.3 Hz), a recycle delay of 2 s and 4000 scans. The optimal ^1^H-^13^C CP contact time resulted 1 ms. The high-power proton decoupling was achieved by the TPPM15 (Time Proportional Phase Modulation) decoupling scheme. Free Induction Decays (FIDs) were processed by Bruker Tospin (v 4.0.1) and MestreC (v. 4.9.9.9, Mestrelab Research) software. Spectra were Fourier Transformed by applying a two-fold zero-filling and adopting an exponential filter function with a line broadening of 200 Hz, prior to being phase- and baseline-corrected.

For the interpretation of ^13^C CPMAS NMR spectra, the overall chemical shift range was divided into the following main resonance regions: alkyl-C (0–45 ppm); methoxyl-C and N-alkyl-C (45–60 ppm); O-alkyl-C (60–110 ppm); aryl-C (110–145 ppm); phenol-C (145–160 ppm) and carboxyl-C (190–160 ppm) [41]. The area of each region was determined by integration (MestreNova 6.2.0 software, Mestrelab Research, Santiago de Compostela, Spain) and expressed as a percentage of the total area. In order to summarize the structural composition of samples, the hydrophobicity index HB/HI was calculated from the relative areas of specific NMR spectral regions as it follows:

HB/HI = ∑ [(0 − 45) + (110 − 160)]/∑ [(45 − 60) + (60 − 110) + (160 − 190)]

#### 3.5.2. Magnetic Resonance Imaging (MRI) Experiments

Spectroscopic MRI experiments were performed at 298 ± 1 K on a 300 MHz (7 Tesla) Bruker Avance wide-bore magnet (Bruker BioSpin, Rheinstetten, Germany) and equipped with a 10 mm μ-imaging MICRO 5 probe working at a ^1^H frequency of 300.13 MHz. Each cylindrical-shaped sample (approximately 10 and 7 mm of height and diameter, respectively) was inserted into a 10 mm NMR tube. Subsequently, it was rehydrated by gently pouring drops of a sodium azide aqueous solution (1 mg mL^−1^) to achieve a visible and homogenous water saturation of the sample (the volume added per sample was 150 μL). Diffusion measurements were performed by using a Pulse Field Gradient (PFG) sequence integrated with stimulated echoes. This experiment was set up with 4 s of recycle delay, 10 scans, δ and Δ diffusion delays of 4 and 13 ms, respectively, and applying gradients at increasing strengths (0, 4.5, 40.3, 112.0, 252.1, 448.1, 757.3, 1295.0, 2800.6 s mm^−2^) in 9 experiments. Since no significant differences in diffusivity were observed by applying gradient pulses either in the longitudinal (*z*) or transverse (*y*) direction, we assumed an isotropic apparent diffusion for all samples. On this basis, the gradients for PFG experiments were applied only in the *z*-direction in all cases. The images consisted of 128 × 128 matrixes and the acquisition geometry applied to acquire each image consisted of 8 interlaced axial slices with a thickness of 1.35 mm, a field of view of 1.33 × 1.2 mm, and 1.3 mm of slice-interspacing. ParaVision 5.1 Bruker software was used to process all MRI data, produce images, and calculate apparent diffusion coefficients.

#### 3.5.3. Scanning Electron Microscopy (SEM)

The morphology of hydrogels was investigated by using a scanning electron microscope (SEM ZEISS EV040). Freeze-dried samples were placed on a stub and sputter-coated with gold-palladium (AGAR sputter COATED B 7340). The SEM observations were performed at magnifications of 10.13 K, with an accelerating voltage of 20.00 kV, at 10 mA current, and 8.5mm < WD < 12.5 mm as a focal length. Several spots were selected and observed for each specimen in order to analyze the microstructure variations.

#### 3.5.4. Rheological Measurements

The viscoelastic properties of hydrogel samples were measured by means of a stress-controlled rotational rheometer (ARG2, TA Instruments) in parallel plates configuration. A Peltier base was used for temperature control. The storage G′ and loss G″ moduli were measured as a function of frequency, ω, in the range 10^−2^–10^1^ Hz. The experimental time was contained to avoid artifacts due to solvent evaporation effects. The tests were carried out in the linear regime, which was previously evaluated for each sample through strain amplitude tests.

#### 3.5.5. Release of Phloroglucinol from Hydrogels

Hydrogels previously loaded with phloroglucinol were immersed in 20 mL of distilled water and the release of the phenolic compound was monitored by UV-Vis spectrophotometry (Perkin Elmer Lambda 25). Water aliquots were sampled periodically at specific time intervals and their absorbance measured at λ_max_ value of 267 nm. In order to maintain the solution concentration, the aliquots were reintroduced in the system after the absorbance reading. The phloroglucinol concentrations were calculated on the basis of a previously-built calibration curve. In particular, UV-Vis absorbance of solutions released from control phloroglucinol-free hydrogels was subtracted from that measured for hydrogels loaded with phloroglucinol, thereby eliminating the contribution by HS and HULIS molecules. Release experiments were performed in triplicate.

The rate of phloroglucinol release from the hydrogel matrices was evaluated by applying the mathematical model proposed by Korsmeyer and Peppas based on the following semi-empirical equation:

M_t_/M_∞_ = k × t^n^

where M_t_ represents the amount of phloroglucinol released at time t; M_∞_ is the total amount of phloroglucinol released at infinite time; k is the rate constant incorporating structural and geometric characteristics of both matrix and phloroglucinol; n is the diffusional exponent that is indicative of the nature of the release mechanism. In the equation above, a value of n = 0.5 indicates a Fickian diffusion mechanism of the active principle from the matrix, while a value 0.5 < n < 1 indicates an anomalous or non-Fickian (diffusion/relaxation) behavior. When n = 1, a case II (erosion/relaxation) transport mechanism is involved, while n > 1 indicates a special case II transport mechanism [38,39].

## 4. Conclusions

We prepared humo-pectic hydrogels by crosslinking, in the presence of calcium ions, pectins of different degrees of methyl-esterification with either HS extracted from cow manure compost or HULIS from depolymerized lignocellulose biorefinery waste. Our results showed that hydrogels composed by pectins only and those obtained by combining pectins and HULIS had a greater capacity to control the release of a model compound than hydrogels prepared by blending pectins and HS. The phloroglucinol release from hydrogels appeared to be influenced by its interactions with the polysaccharidic chains, whose content was larger in hydrogels prepared with pectins only and in those obtained by blending pectins and HULIS than in those made by pectins and HS. Generally, the larger methylation degree of pectic carboxyl groups in HMP-based hydrogels seemed to hinder the formation of hydrogen bonds or other ionic interactions with phloroglucinol hydroxyl groups, thereby favoring the faster diffusion of the phenolic compound out of these hydrogels than from those made with pectin of lesser methylation degree. Therefore, it is suggested that the molecular composition of hydrogels affected not only their morphological and rheological properties but also the rate of phloroglucinol release.

Our results on these humo-pectic hydrogels are potentially useful for sustainable applications in Agriculture since these materials can act as slow-release vectors of agroproducts beneficial to plants, and concomitantly provide both plant biostimulants, such as humic molecules, and metabolic substrate for rhizosphere microorganisms, like pectins.

## Figures and Tables

**Table 1:** Relative carbon distribution (%) over chemical shift regions (ppm) in ^13^C CPMAS NMR spectra of hydrogels containing only low methyl-esterified pectin (H-LMP), high methyl-esterified pectin (H-HMP), or in combination with either humic substances isolated from cow manure compost (H-LMP-HS and H-HMP-HS) or humic-like substances from depolymerized lignocellulose biorefinery residues (H-LMP-HULIS and H-HMP-HULIS).

Sample	Carboxyl-C	Phenol-C	Aryl-C	O-alkyl-C	C-O/C-N	Alkyl-C	HB/HI ^a^
	190–160	160–145	145–110	110–60	60–45	45–0	
H-LMP	10.70	0.00	0.00	81.16	6.99	1.15	0.01
H-HMP	7.30	0.00	0.00	80.14	11.72	0.84	0.01
H-LMP-HS	4.57	0.00	0.78	81.74	10.81	2.11	0.03
H-HMP-HS	2.88	1.09	7.68	66.60	12.11	9.64	0.23
H-LMP-HULIS	4.01	0.00	0.00	82.43	10.95	2.61	0.03
H-HMP-HULIS	0.00	0.00	0.97	85.51	13.52	0.00	0.01

^a^ HB/HI = hydrophobicity index = ∑ [(0 − 45) + (110 − 160)]/∑ [(45 − 60) + (60 − 110) + (160 − 190)].

**Table 2:** Kinetic parameters of phloroglucinol released from hydrogels using the Korsmeyer-Peppas model.

Hydrogels	K ^a^	n ^b^	R^2^
H-LMP	5.7382	0.4072	0.9938
H-HMP	9.3832	0.4225	0.9908
H-LMP-HS	44.4927	0.2851	0.5809
H-HMP-HS	28.3910	0.1990	0.9920
H-LMP-HULIS	2.7121	0.6576	0.9987
H-HMP-HULIS	5.2196	0.5545	0.9953

^a^ Constant which takes into account the structural and geometric characteristics of both the matrix and phloroglucinol. ^b^ Diffusional exponent for phloroglucinol release.
